# Intimal pulmonary artery sarcoma presenting as dyspnea: case report

**DOI:** 10.1186/1477-7800-4-14

**Published:** 2007-06-29

**Authors:** Jeff M Hsing, Snehal G Thakkar, Ernest C Borden, George T Budd

**Affiliations:** 1 Department of Internal Medicine, Taussig Cancer Center, Cleveland Clinic, Cleveland, Ohio, USA; 2 Department of Hematology and Medical Oncology, Taussig Cancer Center, Cleveland Clinic, Cleveland, Ohio, USA

## Abstract

**Background:**

We report a case of pulmonary sarcoma which is a rare cause of the common symptom of dyspnea.

**Case presentation:**

A fifty-one year old previously healthy male presented to the emergency room with complaints of dyspnea on exertion. A cardiac workup including an exercise stress test was negative but an echocardiography showed pulmonary stenosis. Cardiac MRI showed a large mass extending from the pulmonic valve to both the right and left pulmonary arteries suggestive of sarcoma. A complete resection and repair of the pulmonary artery was done and adjuvant chemotherapy with doxorubicin and ifosfamide was recommended. The patient is currently disease free after eighteen months.

**Conclusion:**

Pulmonary artery sarcomas are a difficult diagnosis. The diagnosis may remain elusive for some time until the proper imaging techniques are utilized to make a diagnosis. Earlier and accurate diagnosis may lead to earlier interventions and improve survival.

## Background

Dyspnea is a frequent presentation to the emergency room. The differential diagnosis is extensive and often the diagnosis remains obscure. Pulmonary sarcomas are a rare cause of dyspnea, but do present as such and are often initially misdiagnosed.

## Case Presentation

**Figure 1:**
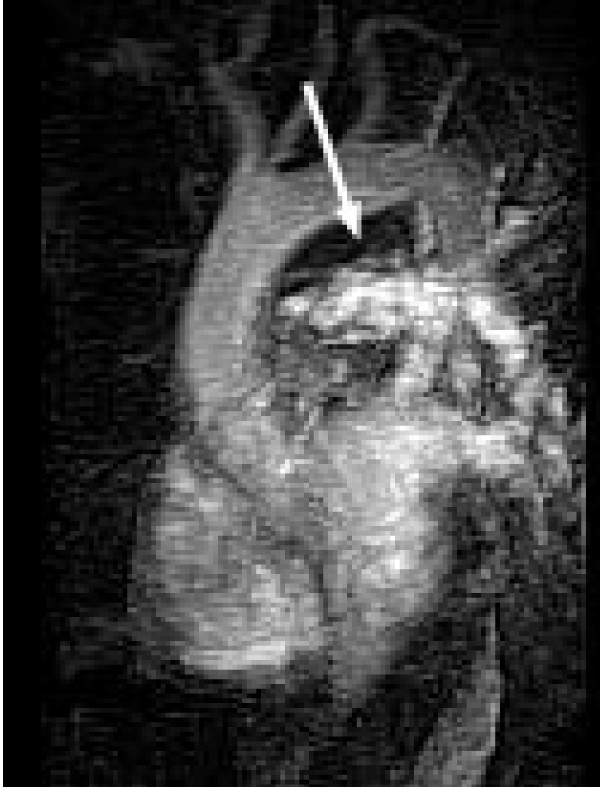
Cardiac MRI showing the sarcoma arising from right ventricle outflow tract to both the right and left pulmonary artery.

**Figure 2:**
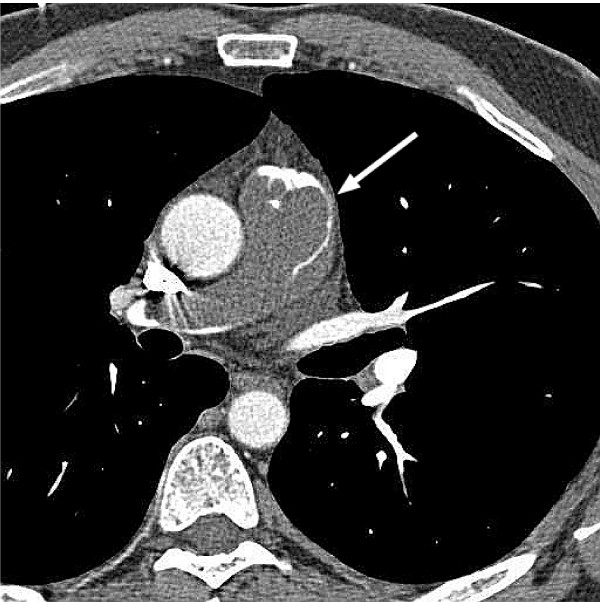
CT scan showing extensive involvement of the main pulmonary artery and into the right trunk.

A 51 year old previously healthy male presented to the emergency room with complaints of dyspnea, chest pain and palpitations while playing tennis. This was the second time over the last month that he experienced some palpitations and dyspnea with exertion. Previously, the patient was ruled out for a myocardial infarction and had a stress echocardiogram which showed mild/moderate pulmonic stenosis, normal right ventricle, and thickened pulmonic valve with doming, but otherwise normal stress test. He was sent home from the emergency room with follow-up with his primary care physician and cardiologist. His cardiologist ordered a cardiac MRI to further evaluate the pulmonic valve. The MRI revealed a large mass starting near the pulmonic valve and extending into the main pulmonary artery and 4 cm into the right pulmonary artery and 3 cm into the left pulmonary artery (Fig 1). The MRI findings were suspicious for a pulmonary artery sarcoma. The patient was admitted into the hospital for further evaluation of the mass. Although the patient's vital signs were stable with normal oxygen saturation he was started on heparin due to suspicion of a thromboembolism. CT scan of the chest was done which showed the large pulmonary artery mass and 2 small 9 mm nodular densities of the right lung without lymphadenopathy (Fig 2). PET-FDG showed uptake in the pulmonary artery trunk, but the 2 nodules did not show abnormal FDG uptake. Cardiothoracic surgery was consulted and the pulmonary artery mass and 2 lung wedge resections were completely excised. Frozen section evaluation was consistent with sarcoma.

**Figure 3:**
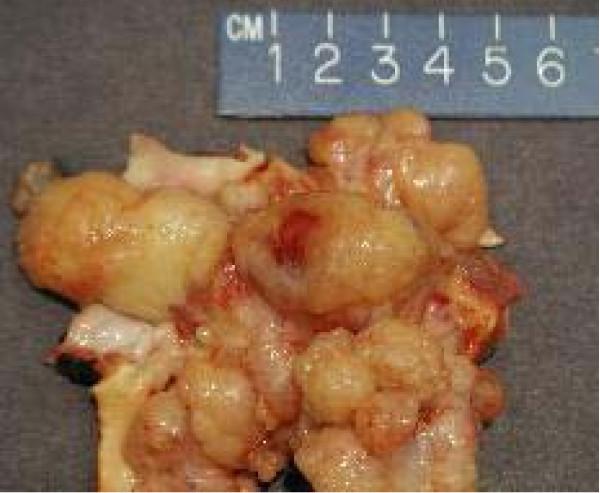
Gross pathology of the sarcoma invading the pulmonary artery.

**Figure 4:**
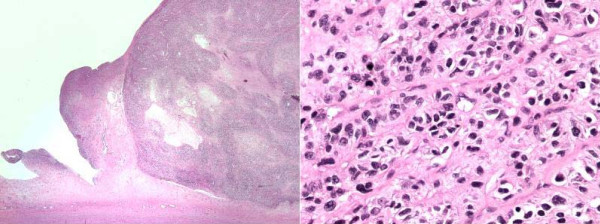
Low field and high field microscopic findings showing spindle cells with hyperchromatic nuclei in myxoid background.

Gross pathology showed a large intimal sarcoma filling the pulmonary artery (Fig 3). It infiltrated the arterial media without extension into the adventitia. The neoplasm consisted of spindled and stellate-shaped cells deposited in variably myxoid stroma (Fig 4). The 2 lung wedges were positive for metastatic sarcoma.

The patient tolerated the surgery well and was discharged with oncology follow-up as an outpatient. Adjuvant chemotherapy with doxorubicin and ifosfamide was initiated soon thereafter. Eighteen months later and after 7 cycles of chemotherapy, he continues to remain disease free by imaging.

## Conclusion

Sarcoma of the great vessels are rare and are usually found in the aorta (most often abdominal), inferior vena cava, or pulmonary artery. Typically, these sarcomas are rare and highly lethal that previously were diagnosed during surgery or autopsy. Patients typically present between the ages of 22 and 81 with female sex predominance. The prognosis is poor with a mean survival of 12 months after onset of symptoms and 1 and 2 year survival rates of 22% and 7% respectively[1]. The presentation usually corresponds to the location of the sarcoma. In the case of a pulmonary artery sarcoma dyspnea, chest pain, cough and hemoptysis are the most frequent complaints. A common error involves diagnosing a pulmonary thromboembolism and treating it as such. There have been a number of case reports and several literature reviews discussing the findings of a pulmonary artery sarcoma. Older case reports usually only made the correct diagnosis during surgery or autopsy and only after a prolonged period of symptoms[2]. More recent advances in imaging technology with multi-slice CT and MRI have enabled better tissue and anatomy characterization [3-5]. This has enabled making the correct diagnosis earlier and differentiating it from other pulmonary vascular pathologies, namely thromboembolism. Quicker diagnosis allows earlier potential curative treatment with surgical resection and adjuvant chemotherapy with or without radiation. At present it is unclear whether earlier diagnosis followed by earlier initiation of treatment leads to improved overall survival.

The World Health Organization (WHO) classify sarcomas on the basis of hematoxylin-eosin, Masson-trichrome, and periodic acid-Schiff stained sections[6]. Sarcomas of the great vessels are usually one of three types: angiosarcoma, leiomyosarcoma, or intimal sarcoma. Leiomyosarcomas arise from the smooth muscle, are spindle cell shaped and stain for desmin and actin. Angiosarcomas arise from the endothelial cells of blood vessels and stain for filament proteins vimentin, factor VIII, and CD34. Sarcomas that don't fit into one of the other categories are termed "intimal." The histologic and immunohistochemical findings are summarized in Table 1. Pulmonary artery sarcomas often arise from the intimal layer of the pulmonary trunk and spread either antegrade into the pulmonary artery branches or retrograde into the pulmonic valve and right ventricle[7]. Metastases are usually found in the lungs, but other sites include kidney, brain, lymph node and skin[6].

**Table 1:** Summary of the common types of sarcomas of the great vessels

**Histologic Type**	**Cell of Origin**	**Histologic feature**	**Immunohistochemical**
*Angiosarcoma*	Blood vessels	Endothelial cells	Vimentin, Factor VIII, CD 34
*Leiomyosarcoma*	Smooth muscle	Spindle shape	Desmin, Actin
*Intimal*	Unknown	Undifferentiated cells	Varies

The mainstay of treatment for sarcoma is surgical resection as this remains the only potentially curative modality. Adjuvant radiation and chemotherapy can be considered following surgical excision although their role remains undefined. An approximate 20% response rate can be expected with a combination chemotherapy regimen involving an anthracycline and an alkylating agent, however, the value of this regimen in the adjuvant setting for pulmonary artery sarcoma is unclear[8,9].

## Abbreviations

CT – Computed Tomography

MRI – Magnetic Resonance Imaging

PET – Positron Emission Tomography

FDG – Fluorodeoxyglucose

## Competing interests

The author(s) declare that they have no competing interests.
